# An Improved Point Cloud Descriptor for Vision Based Robotic Grasping System

**DOI:** 10.3390/s19102225

**Published:** 2019-05-14

**Authors:** Fei Wang, Chen Liang, Changlei Ru, Hongtai Cheng

**Affiliations:** 1 Faculty of Robot Science and Engineering, Northeastern University, Shenyang 110169, China; 1700951@stu.neu.edu.cn; 2 College of Information Science and Engineering, Northeastern University, Shenyang 110819, China; 1870652@stu.neu.edu.cn; 3 School of Mechanical Engineering and Automation, Northeastern University, Shenyang 110819, China; chenght@me.neu.edu.cn

**Keywords:** vision-guided robotic grasping, object recognition, pose estimation, global feature descriptor, iterative closest point

## Abstract

In this paper, a novel global point cloud descriptor is proposed for reliable object recognition and pose estimation, which can be effectively applied to robot grasping operation. The viewpoint feature histogram (VFH) is widely used in three-dimensional (3D) object recognition and pose estimation in real scene obtained by depth sensor because of its recognition performance and computational efficiency. However, when the object has a mirrored structure, it is often difficult to distinguish the mirrored poses relative to the viewpoint using VFH. In order to solve this difficulty, this study presents an improved feature descriptor named orthogonal viewpoint feature histogram (OVFH), which contains two components: a surface shape component and an improved viewpoint direction component. The improved viewpoint component is calculated by the orthogonal vector of the viewpoint direction, which is obtained based on the reference frame estimated for the entire point cloud. The evaluation of OVFH using a publicly available data set indicates that it enhances the ability to distinguish between mirrored poses while ensuring object recognition performance. The proposed method uses OVFH to recognize and register objects in the database and obtains precise poses by using the iterative closest point (ICP) algorithm. The experimental results show that the proposed approach can be effectively applied to guide the robot to grasp objects with mirrored poses.

## 1. Introduction

Three-dimensional (3D) machine vision is a key technology in the field of robotics. Although the rise of 3D vision technology [1,2] is later than two-dimensional (2D) vision technology [3,4], it presents some advantages that 2D vision does not have when performing some complex visual tasks in 3D space. For example, 3D point cloud can provide a wealth of geometric (3D coordinates, curvature variations, surface normals, depth boundaries) and luminosity (color, color variations, transparency, reflectance intensity) information, helping to achieve better results in recognizing objects with less appearance information (e.g., textureless objects) and directly estimating the full 6 degrees of freedom (DOF) object pose. In addition, under unfavorable lighting conditions, 3D data provided by infrared laser technology can achieve better results than 2D images. In recent years, low-cost real-time 3D sensors such as Microsoft Kinect and Asus Xtion have become low-cost consumer devices accessible to ordinary users. These sensors can be used to generate color 3D point clouds on the surface of a given scene in real time, which also promotes the research of 3D object recognition and registration. 

For 3D point cloud, there are two kinds of object recognition methods: local feature descriptors [5,6,7] and global feature descriptors [8,9,10,11,12]. Global feature descriptors describe the geometry, appearance or both of the object point cloud, which is more advantageous in object recognition and pose estimation [13]. Moreover, compared with local methods, global methods compute less descriptors and have a simpler recognition pipeline to speeding up the recognition speed [14], which is very important for applications designed to run in nearly real time [15]. The viewpoint feature histogram (VFH) [8] is a global feature descriptor which can be used for object recognition and pose estimation in a 6DOF robot grasping operation. Previous work has proved the computational efficiency and high recognition ability of this feature [16,17]. However, it is difficult for VFH to distinguish the mirrored poses of the object having the mirrored structure with respect to the viewpoint. Paper [18] proposes a modified viewpoint feature histogram (MVFH), which applies the calculation method of extended FPFH to the calculation of viewpoint component. Although this work is beneficial, it does not theoretically explain the reasons for the effect. In order to solve this difficulty, a novel global feature descriptor named orthogonal viewpoint feature histogram (OVFH) is proposed in this paper. The OVFH uses an extended fast point feature histogram (FPFH) to describe the surface shape of the object. Its viewpoint component is calculated by using the orthogonal vector of the viewpoint direction, which is calculated based on the reference frame of the object point cloud, to obtain the distinguishing ability for the mirrored poses. 

The main contributions of this paper are (1) a novel and efficient global feature descriptor for object recognition and pose estimation; (2) an evaluation of the object recognition rate and the ability to distinguish the mirrored poses; (3) a visual guidance method for a robotic grasping system.

The rest of the paper is organized as follows: Section 2 introduces the proposed improved global feature descriptor OVFH. Section 3 describes the object recognition and pose estimation algorithms for robotic grasping operation. Section 4 details the experimental device and the effectiveness of the proposed algorithm in publicly available datasets and robotic grasping experiments. Finally, conclusions and future work are discussed in Section 5.

## 2. Improved Global Feature Descriptor

In this section, VFH is reviewed and used to derive the proposed OVFH, which is consistent with our goal of performing well in object recognition and distinguishing mirrored poses. The specific calculation procedures are detailed in the following subsections.

### 2.1. Global Feature Descriptor VFH

The object’s global feature descriptor is a high dimensional representation of the object’s 3D shape and is designed for object recognition and pose retrieval. VFH [8] is an effective point cloud feature which is used for the applications about object recognition and 6DOF pose estimation. VFH is a combined histogram containing viewpoint direction features and an extended FPFH [5], which represents the distributions of the four angles representing the geometric characteristics of the point cloud. In point cloud *P*, let ***p****_v_* denote the position of the viewpoint, ***p****_c_* denote the center of gravity of of all points ***p****_i_* in the point cloud, and ***n****_c_* donate the average normal vector of all normal ***n****_i_* at point ***p****_c_*. ***p****_c_* and ***n****_c_* are calculated as follows:

(1) $$p_{c}=\frac{1}{n}\sum_{i=1}^{n}p_{i},$$

(2) $$n_{c}=\frac{1}{n}\sum_{i=1}^{n}n_{i},$$

For each ***p****_i_* in the cloud *P*, a local coordinate system is defined at point ***p****_c_*, as in Equation (3).

(3) $$\left\{ \begin{array}{l} u=n_{c}\\ v=\frac{p_{i}-p_{c}}{{\left\|p_{i}-p_{c}\right\|}_{2}}\times u\\ w=u\times v \end{array}\right.,$$

**Figure 1:**
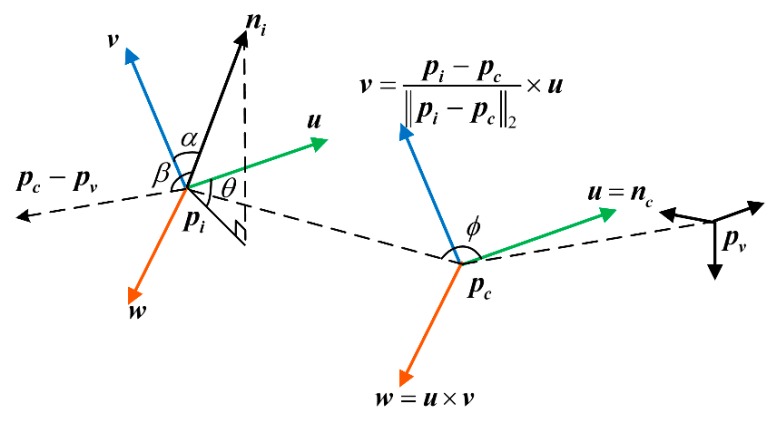
Relative relationship between points described by a set of angles.

Using the local coordinate system ***uwv*** defined above, the relative deviation between the centroid vector ***n****_c_* and the unit normal vector ***n****_i_* of the point ***p****_i_* can be represented by a set of angles, as shown in Figure 1.

The feature descriptor for each point in the point cloud can be represented by a quintuple (*α*, *ϕ*, *θ*, *d*, *β*), which is calculated as follows:

(4) $$\left\{ \begin{array}{l} \cos\alpha =v\cdot n_{i}\\ \cos\beta =n_{i}\cdot \frac{p_{c}-p_{v}}{{\left\|p_{c}-p_{v}\right\|}_{2}}\\ \cos\phi =u\cdot \frac{p_{i}-p_{c}}{d}\\ \theta =\arctan\frac{w\cdot n_{i}}{u\cdot n_{i}}\\ d={\left\|p_{i}-p_{c}\right\|}_{2} \end{array}\right.,$$

**Figure 2:**
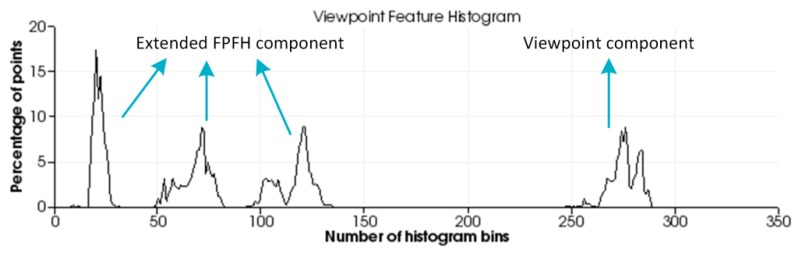
Viewpoint feature histogram.

The percentages of the values of cos*α*, cos*ϕ*, *θ*, *d*, and cos*β* of each point in the point cloud *P* falling in different bins are counted, respectively corresponding to the curves on the abscissa ranges [1, 45], [46, 90], [91, 135], [136, 180], [181, 308] of the feature histogram. Since the distance *d* between points gradually increases along the viewpoint direction and the density of the local points will affect the feature result, *d* is often omitted in the 2.5-dimensional data acquired by the robot for better robustness. Finally, the VFH descriptor describes the point cloud using a total of 263 bins. Figure 2 shows an example of the VFH.

**Figure 3:**
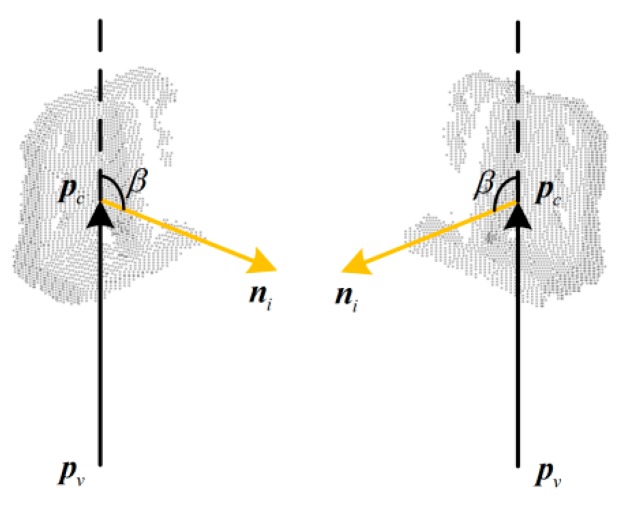
The same viewpoint features of viewpoint feature histograms (VFHs) in mirrored poses.

Although Rusu et al. [8] have obtained promising results in using VFH as a 3D feature for object recognition and 6DOF pose estimation, they have encountered limitations of accurate 3D pose estimation. For example, if the surface of the object has mirror symmetry, it will get similar VFHs in symmetrical poses, as shown in Figure 3. The vector ***n****_i_* is the normal vector at point ***p****_i_*. Although the two poses are different poses mirrored with respect to the viewpoint direction, the two VFHs are highly similar because their surface normals of each point have similar or identical angular deviations from the viewpoint direction. In the case shown in the figure, the kind of object can be correctly identified using VFH, but the mirrored poses are confused.

### 2.2. Improved Global Feature Descriptor OVFH

In order to overcome this drawback, it is necessary to modify the existing VFH descriptor. Chen et al. [18] have did some groundbreaking work in this area and proposed the MVFH descriptor to improve the viewpoint direction component of VFH. The MVFH gives three components to the viewpoint direction component using the method similar to estimating the extended FPFH. However, just as the geometric features characterized by the extended FPFH are very similar in the mirrored poses, the viewpoint direction component counted by this method still cannot explain theoretically how to distinguish the mirrored poses.

The core idea of solving this problem is to change the way to calculate the viewpoint direction component so that statistical angle values are different in the mirrored poses. According to this idea, an OVFH descriptor is proposed, which is described in detail as follows.

First, the reference frame of the point cloud needs to be estimated using the principal component analysis (PCA) method, which will help to compute the orthogonal vector of the viewpoint direction. All the points ***p****_i_* belonging to the point cloud are given to represent the view of the object, where *i* ∈ {1,..., *n*}. Their centroid ***p****_c_* are calculated according to Equation (1) and used as the origin of object reference frame. After that, the covariance matrix *C* of all points is calculated by ***p****_i_* and ***p****_c_* as the following equation:

(5) $$C=\frac{1}{n}\sum_{i=1}^{n}(p_{i}-p_{c}){\left(p_{i}-p_{c}\right)}^{T},$$

**Figure 4:**
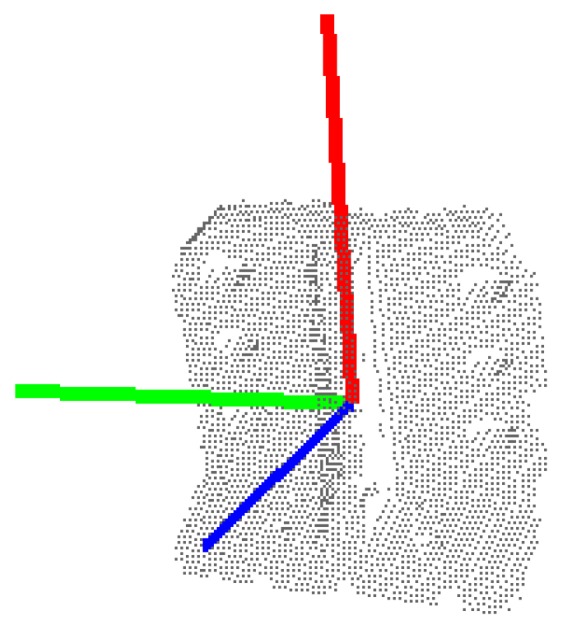
Reference frame estimated from three-dimensional (3D) point cloud: x-axis (red), y-axis (green), and z-axis (blue).

Then, the eigenvalue *λ_j_* of *C* and its corresponding eigenvector ***v****_j_* that satisfy *C**v**_j_* = *λ_j_**v**_j_*, where *j* ∈ {1, 2, 3}, are computed. The eigenvector ***v****_min_* which is corresponding to the minimum eigenvalue *λ_min_* is taken as the z-axis of the reference frame. In order to eliminate the ambiguity in the z-axis direction, if the angle between ***v****_min_* and the observation direction is in the range of [−90°, 90°], the opposite vector of ***v****_min_* is taken. This ensures it points to the observer all the time. The eigenvector ***v****_max_* which is corresponding to the maximum eigenvalue *λ_max_* is taken as the x-axis of the reference frame. After that, the y-axis is computed by ***y*** = ***v****_min_* × ***v****_max_*. The reference frame estimated for a given partial view is shown in Figure 4.

The z-axis pointing to the observer is obtained after determining the reference frame representing the overall point cloud of the partial view. The cross product of the z-axis and the viewpoint direction is calculated by Equation (6) to obtain an orthogonal vector of the viewpoint direction:

(6) $$v_{o}=z\times (p_{c}-p_{v}),$$

**Figure 5:**
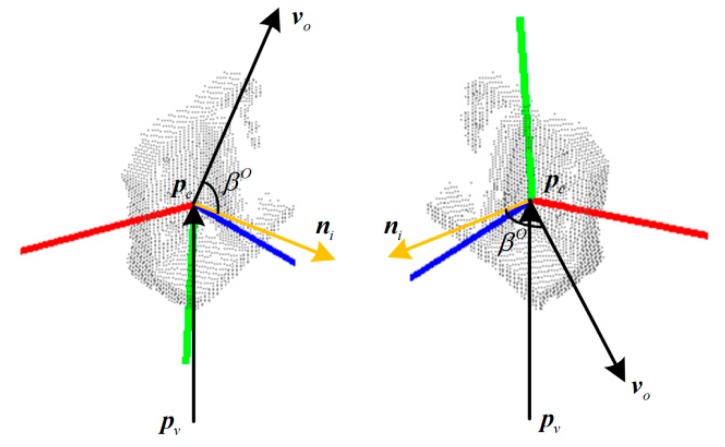
The different viewpoint features of orthogonal viewpoint feature histograms (OVFHs) in mirrored poses.

Different from VFH [8], OVFH calculates the viewpoint component by counting a histogram, which is a statistic of the angles between the orthogonal vector of the viewpoint direction and each normal, so that the viewpoint direction component of the mirrored poses are different from each other, as shown in Figure 5. The angular deviation cos*β_O_* between the orthogonal viewpoint vector and each normal ***n****_i_* is calculated by Equation (7):

(7) $$\cos\beta_{O}=n_{i}\cdot \frac{v_{o}}{{\left\|v_{o}\right\|}_{2}},$$

Using the speed and discriminative power of the FPFH to ensure the strong recognition result of the OVFH, the FPFH is extended to estimate the entire object point cloud. The difference between the normal ***n****_i_* of each point ***p****_i_* and the central normal ***n****_c_* can be represented by cos*α_O_*, cos*ϕ*_O_, and *θ_O_*, which represent relative pan, tilt, and yaw angles, respectively. They are given by the following equations:

(8) $$\left\{ \begin{array}{l} \cos\alpha_{O}=v\cdot n_{i}\\ \cos\phi_{O}=u\cdot \frac{p_{i}-p_{c}}{{\left\|p_{i}-p_{c}\right\|}_{2}}\\ \theta_{O}=\arctan\frac{w\cdot n_{i}}{u\cdot n_{i}} \end{array}\right.,$$

**Figure 6:**
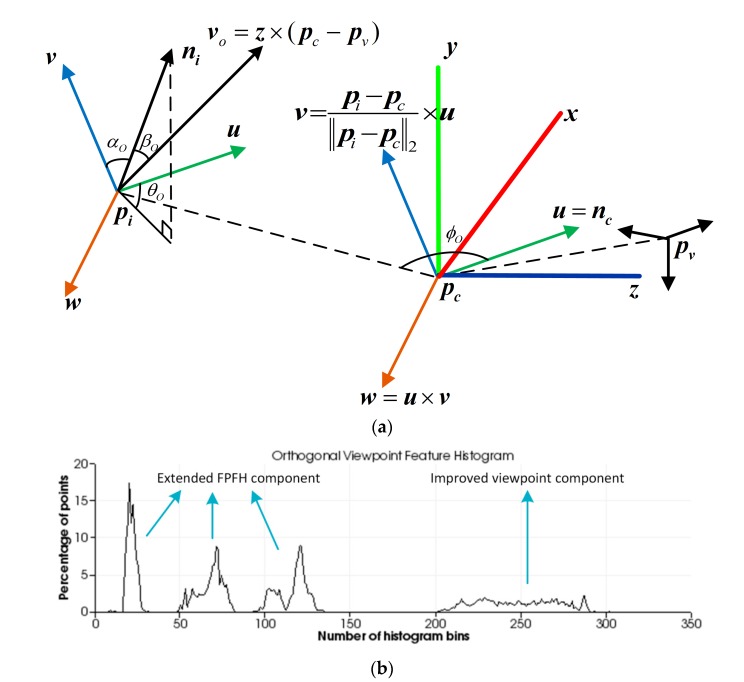
OVFH concept map: (**a**) improved feature description; (**b**) orthogonal viewpoint feature histogram.

**Figure 7:**
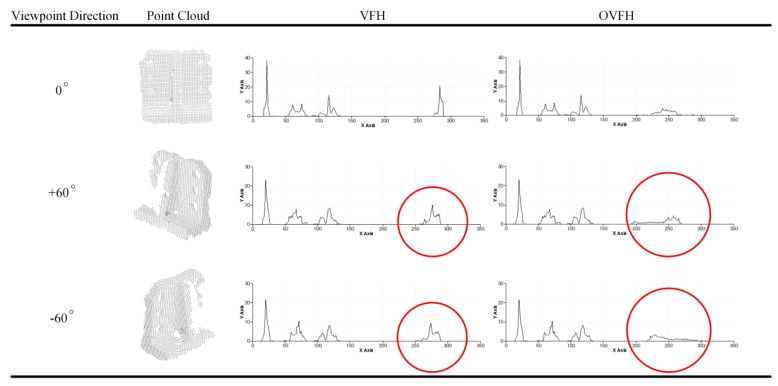
Comparison of two feature descriptors.

In summary, the proposed OVFH descriptor contains two components: one is the surface shape component constituted of the extended FPFH, and the other is the viewpoint component improved by the orthogonal vector of the viewpoint direction. The OVFH uses 45 bins for each value of the extended FPFH by default, and another 128 bins for the improved view component, thus, the OVFH descriptor has 263 dimensions. Figure 6 shows the principle and result of OVFH. Figure 7 shows the calculation results of VFH and OVFH in the cases that the object faces the viewpoint and deviates from the viewpoint direction by +60° and –60° yaw angle, respectively. As shown in the figure, although both VFH and OVFH assign 128 bins to encode the viewpoint direction component, the viewpoint direction information of VFH is only distributed in 64 bins. This is because the normals of the point cloud always point to the sensor, and their dot product with the central viewpoint direction must be in the range [0, 1]. The dot product of the point cloud normals and the orthogonal vector of the central viewpoint direction is in the range of [−1, 1]; thus, the viewpoint component of OVFH is distributed in all 128 bins and reserves more viewpoint information than VFH. The VFHs of mirrored poses are very similar, which may cause their corresponding poses are misidentified because of the similar match scores in the template matching phase. The OVFH descriptors of mirrored poses have distinctly different viewpoint direction components in the mirrored poses, so that the false mirrored pose can be avoided in the template matching phase.

## 3. Visual Guidance Algorithm for the Robotic Grasping System

**Figure 8:**
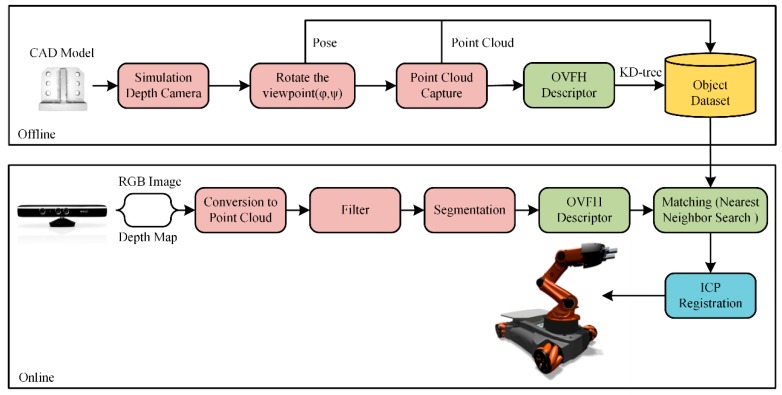
Algorithm for the robotic grasping system.

The robotic visual grasping algorithm includes two phases, offline and online, as shown in Figure 8. In the offline phase, a database that has complete poses of experimental objects is created by changing the sensor viewpoint. The object poses under each viewpoint are combined with the available grasping poses to teach the robot how to grasp the object in different poses. After that, all relevant information is stored in the database, including point clouds, feature descriptors, classification information, and grasping poses for each viewpoint. In the online phase, the scene point cloud is captured using a depth camera. After filtering and segmenting the scene point cloud, the global feature descriptor of the object is calculated to match the database. And the recognition result is the sample that has the most similar feature histogram with the object. Finally, the iterative closest point (ICP) algorithm [19] is used to calculate the precise pose to generate the trajectory and pose for robotic grasping operation.

### 3.1. Creation of the Database

The database mainly consists of two parts: a multi-view point cloud database and a grasping pose database. 

A multi-view point cloud database is created to contain all the poses of the object to be grabbed. Point cloud data from different perspectives can be captured by building a rotating platform such as [8], but creating a training database for a reasonable number of objects using a real device can be a cumbersome task, and even difficult if one wants to have all the different views and poses of an object. Alternatively, as described in [9], if the object has an available 3D computer aided design (CAD) model, a virtual camera can be placed directly around the object in the rendering system and all desired viewpoints can be obtained without calibrating the system and a time-consuming capture process. This is a database creation method which is low-cost and easy to extend object set; therefore, this paper uses this method to create a database. 

**Figure 9:**
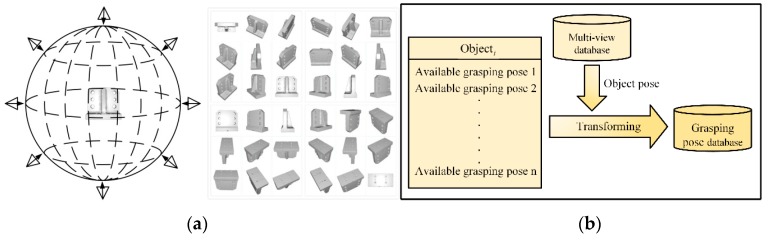
Database creation: (**a**) multi-view point cloud database; (**b**) grasping pose database.

Taking CAD model as the center of the sphere, the bounding sphere with radius *r* is established. A virtual depth camera [20] is set up on the viewpoints uniformly selected on the spherical surface to capture the object point clouds. As shown in Figure 9a, first, to ensure the coverage and uniformity of viewpoint selection, viewpoints are selected on the bounding sphere at every 15° yaw angle and pitch angle, denoted as (φ,ψ). Then, the virtual depth camera is set up at each viewpoint to capture the corresponding object point cloud, and record the pose data of the object model in the camera coordinate system. Finally, the point cloud data is processed offline. The OVFH descriptors of the point clouds under each viewpoint are calculated and the feature files are saved in the database. 

Objects usually have multiple stable poses. It is impossible for the robot to grip the object in the same grasping pose in any cases because of the limitation of the environment and robot’s working space. Therefore, it is necessary to teach the robot how to grab objects in different poses. A plurality of stable robot grasping poses relative to the object are recorded in the database, and the robot grasping poses in different object poses can be obtained by rotating and transforming the object poses under different viewpoints in the multi-view point cloud database. Figure 9b shows the database that records the grasping poses. 

### 3.2. Object Recognition and Pose Estimation

**Figure 10:**
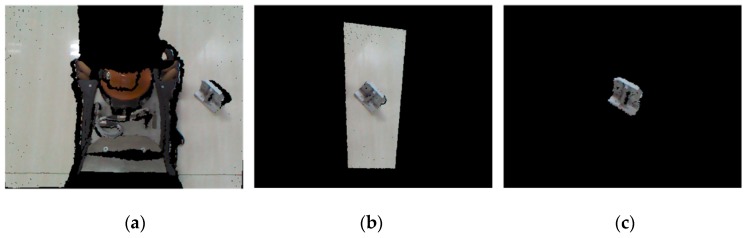
Point cloud pre-processing: (**a**) scene point cloud; (**b**) passthrough filter; (**c**) object segmentation.

A 640 × 480 pixels RGB-D image captured by the Kinect v1 sensor is processed with the PCL library and converted into a point cloud containing 307,200 points (see Figure 10a). It requires initial filtering before the segmentation process. First, invalid points (NaN) that are useless for 3D processing without depth information due to factors such as specular surface, occlusion or transparency are removed. Then, a passthrough filter is used to remove all the points located outside the defined range. Experiments have shown that it is impossible to identify small objects reliably outside the range of 0.4–1.5 m away from the sensor. Therefore, the cut-off distance of the passthrough filter along the Z-axis is set as this range. The appropriate X and Y axis ranges are set to confine the isolated region of interest (ROI) to the graspable workspace of the robotic arm (see Figure 10b). Then, the random sample consensus (RANSAC) algorithm is used to detect the principal plane of the remaining point cloud and remove the inlier points of the plane, that is, to remove the ground points. Finally, the cluster of the target object is obtained by Euclidean cluster segmentation, and the noise clusters are eliminated by setting an appropriate threshold of the number of the cluster points (see Figure 10c). 

**Figure 11:**
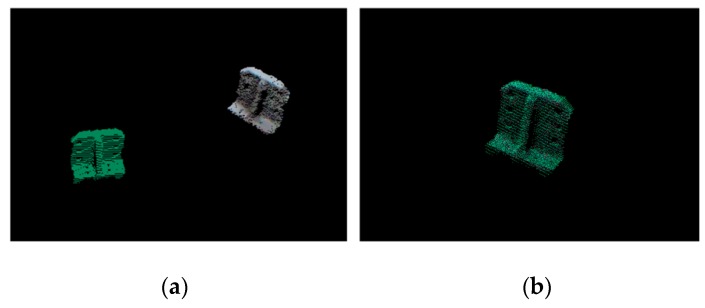
Registration results: (**a**) Initial relative pose; (**b**) Refined pose after the iterative closest point (ICP).

The OVFH feature of the target point cloud is calculated after the target point cloud in the scene is obtained through the above preprocessing. The obtained OVFH descriptor is compared with the multi-view point cloud database by the k-nearest neighbor search based on K dimension (K-D) tree, and the winning result is selected as the object recognition result with rough pose estimation. Then, the point cloud of winning pose is translated to the centroid of the target object and iteratively optimized by ICP algorithm. This algorithm iteratively modifies the rigid transformation matrix between two point clouds to minimize their distance until the iteration error is less than the threshold or the current iteration number is greater than the maximum number. Figure 11 shows the registration effect of the template point cloud and the target point cloud. Finally, the robotic manipulator grasps the object based on the object refined pose after using ICP.

## 4. Experimental Results

### 4.1. Experimental Results on the Data Set

**Figure 12:**
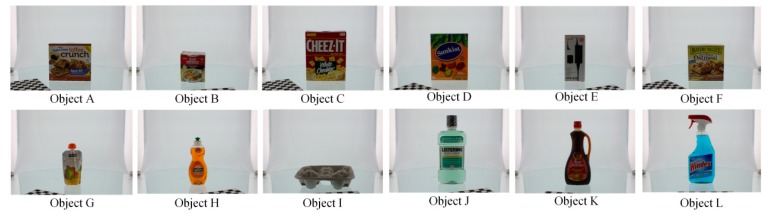
12 tested objects.

**Figure 13:**
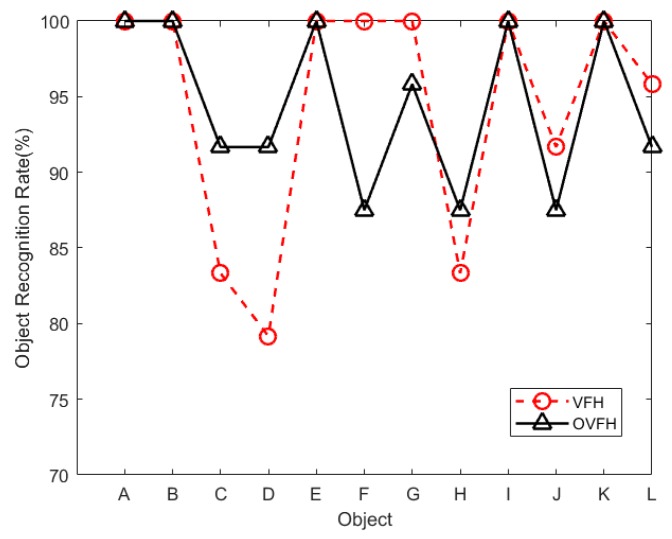
Object recognition rate.

**Figure 14:**
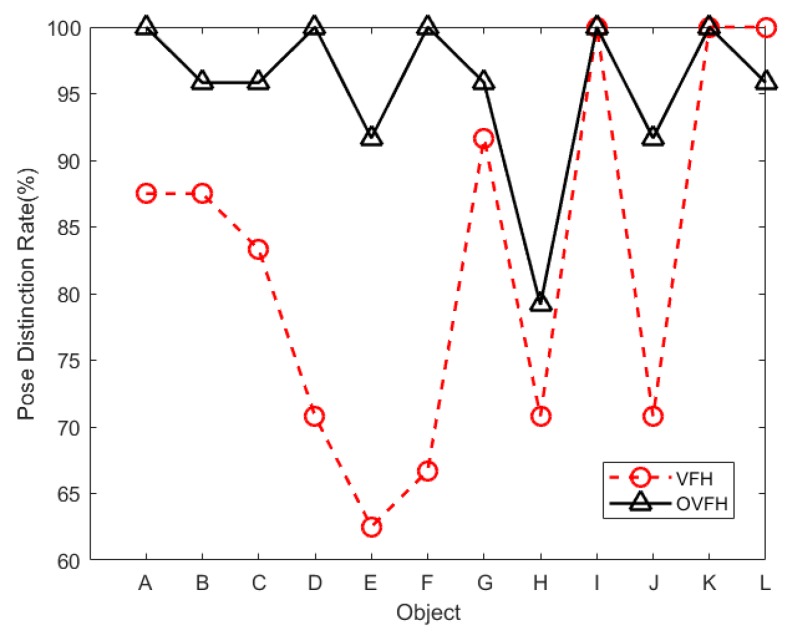
Mirrored pose distinction rate.

The hardware used in the evaluation experiment was a computer with Intel Core i5-7500 CPU @ 3.40 GHz processor and 16 GB RAM. In order to prove that the proposed descriptor OVFH is improved in pose retrieval compared with VFH, a publicly available dataset [21] was used for test experiments. Each object in the data set has 600 point clouds which are captured from five polar angles and 120 turntable positions, with an azimuth equidistance of 3°. The 12 objects shown in Figure 12 were selected from the BIGBIRD data set for a comparative experiment of pose retrieval using VFH and OVFH. A complete pose database (12 ∗ 5 ∗ 24 = 1440 in total) was created by selecting poses at 24 equally spaced 15° azimuths from each polar angle of each object. In order to verify the pose identification ability of OVFH and VFH, 24 point clouds with mirrored poses of each object were selected as the test set. Figure 13 shows the object recognition accuracy using two kinds of feature descriptors. As far as the test was concerned, OVFH had similar accuracy to VFH in object recognition. The average recognition rates of OVFH and VFH were both 94.44% for all 12 objects. Figure 14 shows the mirrored pose distinction accuracy using two kinds of feature descriptors. For mirrored poses, the average distinction rate of OVFH (95.49%) was significantly higher than that of VFH (82.6%). Table 1 presents the computation time for each procedure when using two kinds of descriptors to test. Although OVFH descriptor required an additional step of reference frame estimation, OVFH could avoid false pose recognition and provide a better initial pose for ICP, which greatly reduced the time consuming of refining pose. Table 1 shows that the average computation time was 856.866 ms when object recognition and rough pose estimation were performed using VFH and the pose was further refined using ICP. OVFH reduced the average computation time to 546.212 ms because reducing the number of ICP iterations. The distance root mean squared error (RMSE) of corresponding point pairs in the point cloud registration process is used to describe the error of the pose estimation. It can be seen from the Table 1 that OVFH can obtain higher average precision of pose estimation by providing better initial values for ICP. From Figure 13 and Figure 14 and Table 1, OVFH enhances the distinction capability of the mirrored pose while preserving the identification capability of VFH, and is more computationally efficient and precise in the accurate pose estimation combined with the ICP. Experimental results show that the proposed feature (OVFH) can be used to improve the performance of pose retrieval. 

### 4.2. Robotic Grasping Experiment

**Figure 15:**
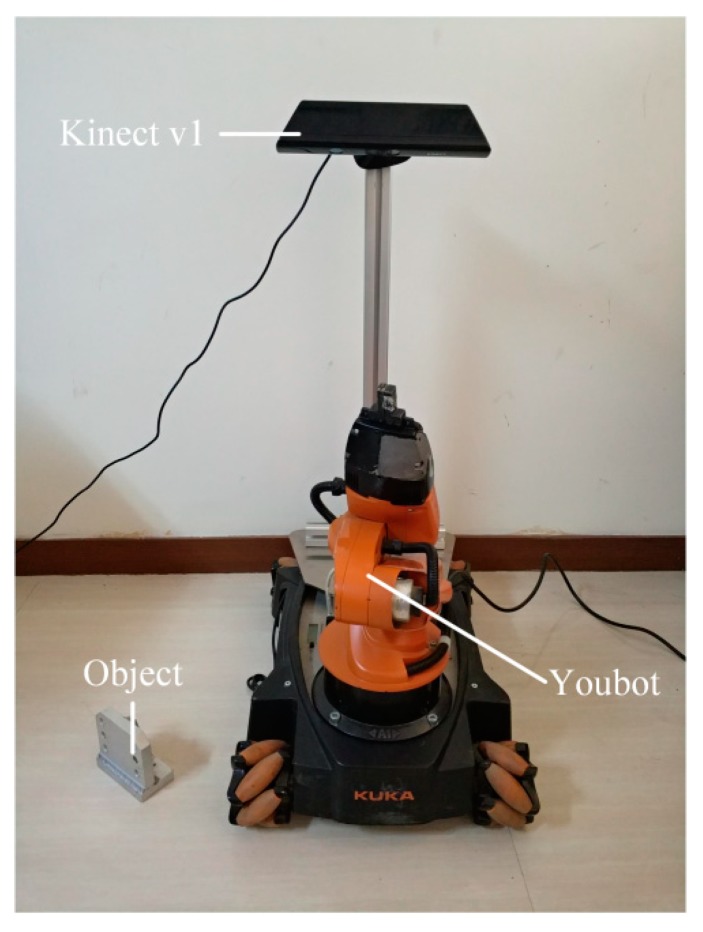
The hardware setup of the robotic grasping experiment.

**Figure 16:**
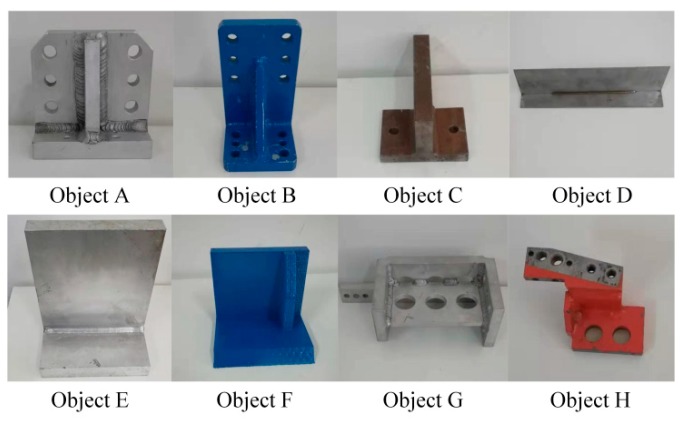
The objects used in robotic grasping experiment.

Figure 15 shows the hardware setup of the robotic grasping experiment. A KUKA Youbot robot was used to grasp objects, and Microsoft Kinect v1 was mounted on the robot’s stand to capture point clouds for robotic vision. The computer used for object recognition and pose estimation was equipped with an Intel i5 CPU and 16 GB RAM. Eight objects used for the grasping experiment are shown in Figure 16, where objects A-E have mirrored structure. A multi-view point cloud database was built by the CAD models of all experimental objects, and the robot was taught how to capture objects of different poses.

**Figure 17:**
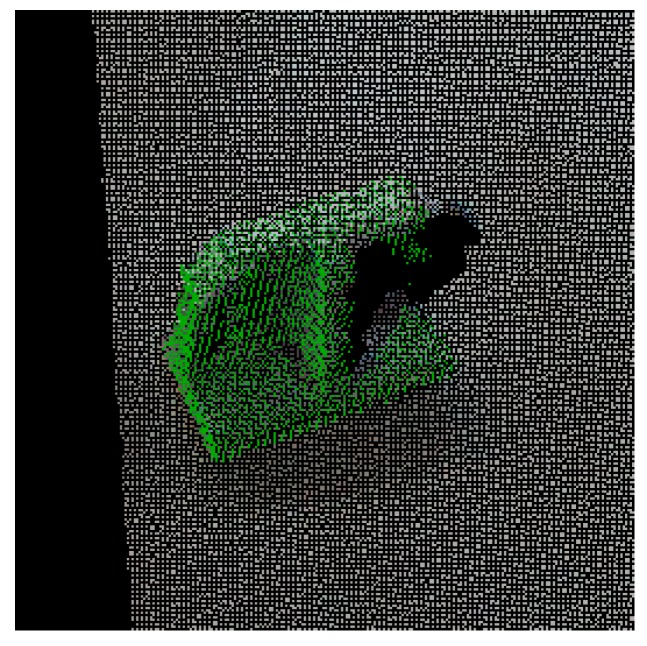
The result for object recognition and registration.

**Figure 18:**
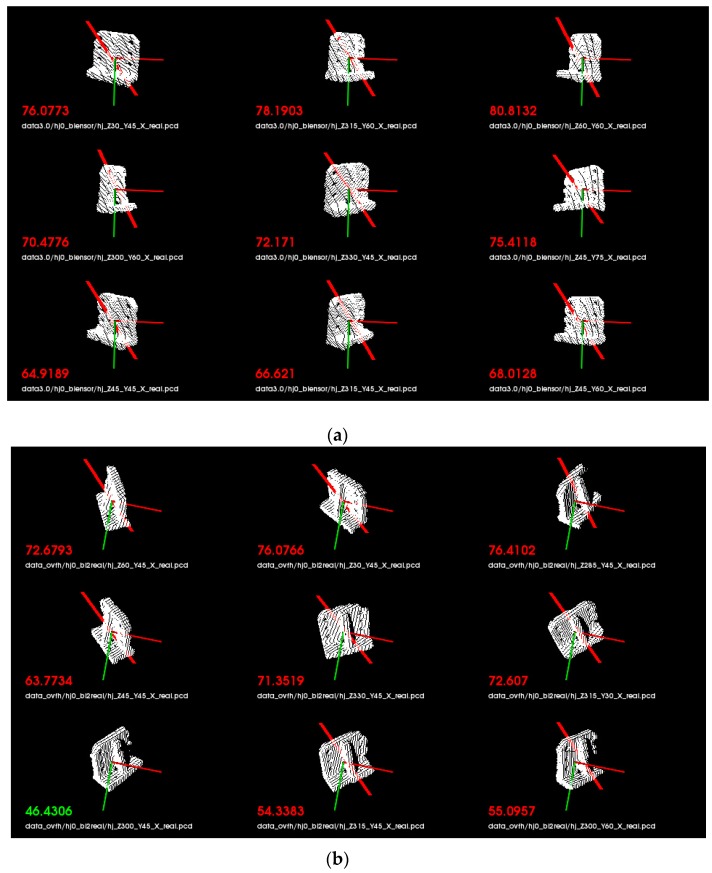
Matching results for (**a**) VFH descriptor and (**b**) OVFH descriptor.

**Figure 19:**
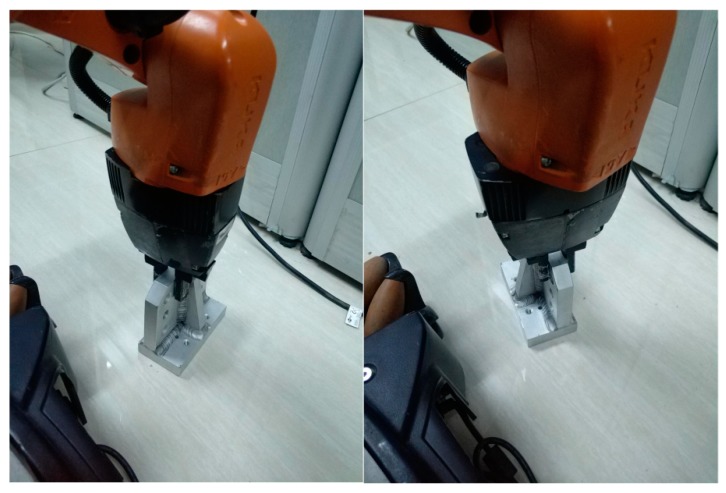
The grasping results for object with mirrored poses.

Experiments were performed on eight objects, each of which was set to 10 initial poses in the robot’s workspace. Figure 17 shows the results of object recognition and pose estimation. As shown in the figure, the point cloud collected online was precisely registered with the recognition result in database. To test the effectiveness of the proposed OVFH descriptor, the first nine matching scores were used to determine the distinction capability of the mirrored poses, as shown in Figure 18. Figure 18a,b, respectively, show the results of object recognition using the VFH and OVFH descriptor, when the object A was placed in a pose of −60° relative to the viewpoint. The result in the lower left corner was the best match. When using VFH, the true pose and its mirrored poses were alternately arranged, and a false positive was generated for the mirrored pose. When using OVFH, the true pose was correctly identified, and the matching scores of the mirror poses and the true pose were quite different, which would avoid the false positive effectively. The experimental results of eight objects are recorded in Table 2. For objects F-H without mirrored structure, VFH and OVFH exhibited similar performance. For objects A-E with mirrored structure, VFH could not distinguish its mirror poses. Identifying the wrong initial pose would increase the registration time of ICP and the pose estimation error, and even lead to registration failure. Since OVFH avoided false pose Identification, the convergence was faster when using ICP to refine the pose, and the average computation time was reduced to 0.523 s. At the same time, correct pose recognition made the model point cloud and the target point cloud better fit, thus obtaining higher pose estimation accuracy. After object recognition and registration, the refined pose was used to guide the robot to grasp the object. Figure 19 shows the grasping experiment results of an object with mirrored poses. 

## 5. Conclusions and Future Work

In order to correctly distinguish the mirrored poses relative to the viewpoint, an effective global feature descriptor OVFH is proposed in this paper, which was successfully applied to object recognition and pose estimation. The proposed method computes an orthogonal vector of the viewpoint direction by using a reference frame estimated for the entire point cloud. This orthogonal vector is used to improve the viewpoint component of the feature descriptor. Experimental results in public data set show that OVFH descriptor can characterize object poses well and enhance the ability to distinguish mirrored poses. Based on OVFH descriptor, an object recognition and pose estimation method for vision-guided robotic grasping system is designed. The experimental results show that the proposed vision-guided robotic grasping method can effectively distinguish the mirrored poses and guide the robot to grasp different objects. 

For future work, the proposed feature descriptor can be extended by color description to obtain the ability to recognize objects with the same geometries and different patterns. It will also be studied to combine the proposed idea of calculating orthogonal viewpoint direction with other feature descriptors to obtain better results.

## Figures and Tables

**Table 1:** Computation time for processing a single object instance.

Method	Procedure	Average Time (ms)	Average RMSE (m)
VFH + ICP	Description	1.694	3.219 × 10^−5^
	Pose refinement	855.172	
OVFH + ICP	Reference frame estimation	10.767	1.391 × 10^−5^
	Description	1.832	
	Pose refinement	533.613	

**Table 2:** Experimental results of eight objects.

Object	VFH + ICP				OVFH + ICP		
	Pose Distinction Rate (%)	Average Time (s)	Average RMSE (m)		Pose Distinction Rate (%)	Average Time (s)	Average RMSE (m)
A	60	0.878	3.388 × 10^−5^		100	0.428	1.543 × 10^−5^
B	70	0.973	6.895 × 10^−5^		100	0.574	2.258 × 10^−6^
C	90	0.676	1.674 × 10^−5^		100	0.471	2.852 × 10^−6^
D	70	0.747	1.044 × 10^−5^		90	0.338	2.994 × 10^−6^
E	80	1.079	6.611 × 10^−5^		100	0.775	2.415 × 10^−6^
F	100	0.501	4.317 × 10^−6^		100	0.494	4.062 × 10^−6^
G	100	0.672	6.213 × 10^−6^		100	0.647	6.429 × 10^−6^
H	100	0.446	5.379 × 10^−6^		100	0.457	5.240 × 10^−6^
Average Value	83.75	0.747	2.650 × 10^−5^		98.75	0.523	5.210 × 10^−6^
